# Influence of Femtosecond Laser Modification on Biomechanical and Biofunctional Behavior of Porous Titanium Substrates

**DOI:** 10.3390/ma15092969

**Published:** 2022-04-19

**Authors:** Ana M. Beltrán, Mercè Giner, Ángel Rodríguez, Paloma Trueba, Luisa M. Rodríguez-Albelo, Maria Angeles Vázquez-Gámez, Vanda Godinho, Ana Alcudia, José M. Amado, Carmen López-Santos, Yadir Torres

**Affiliations:** 1 Departamento de Ingeniería y Ciencia de los Materiales y del Transporte, Escuela Politécnica Superior, Universidad de Sevilla, 41011 Seville, Spain; abeltran3@us.es (A.M.B.); ptrueba@us.es (P.T.); vfortio@us.es (V.G.); ytorres@us.es (Y.T.); 2 Departamento de Citología e Histología Normal y Patológica, Universidad de Sevilla, 41009 Seville, Spain; mginer@us.es; 3 Departamento Ingeniería Naval e Industrial, Escuela Politécnica Superior, Campus Industrial, Universidade da Coruña, 15403 Ferrol, Spain; jose.amado.paz@udc.es; 4 Departamento de Medicina, Universidad de Sevilla, 41009 Seville, Spain; mavazquez@us.es; 5 Departamento de Química Orgánica y Farmacéutica, Facultad de Farmacia, Universidad de Sevilla, 41005 Seville, Spain; aalcudia@us.es; 6 Departamento de Física Aplicada I, Escuela Politécnica Superior, Universidad de Sevilla, 41011 Seville, Spain; mlopez13@us.es; 7 Nanotecnología en Superficies y Plasma, Instituto de Ciencia de Materiales de Sevilla, 41092 Seville, Spain

**Keywords:** porous titanium, femtosecond laser, surface modification, instrumented micro-indentation, scratch test, wettability, cellular behavior

## Abstract

Bone resorption and inadequate osseointegration are considered the main problems of titanium implants. In this investigation, the texture and surface roughness of porous titanium samples obtained by the space holder technique were modified with a femtosecond Yb-doped fiber laser. Different percentages of porosity (30, 40, 50, and 60 vol.%) and particle range size (100–200 and 355–500 μm) were compared with fully-dense samples obtained by conventional powder metallurgy. After femtosecond laser treatment the formation of a rough surface with micro-columns and micro-holes occurred for all the studied substrates. The surface was covered by ripples over the micro-metric structures. This work evaluates both the influence of the macro-pores inherent to the spacer particles, as well as the micro-columns and the texture generated with the laser, on the wettability of the surface, the cell behavior (adhesion and proliferation of osteoblasts), micro-hardness (instrumented micro-indentation test, *P–h* curves) and scratch resistance. The titanium sample with 30 vol.% and a pore range size of 100–200 μm was the best candidate for the replacement of small damaged cortical bone tissues, based on its better biomechanical (stiffness and yield strength) and biofunctional balance (bone in-growth and in vitro osseointegration).

## 1. Introduction

Today, the demand for implants obtained from natural and synthetic biomaterials for different parts of the human body is exponentially increasing. Within metallic biomaterials, titanium (Ti) and its alloys are considered one of the best choices for the manufacture of dental and bone implants due to their acceptable biomechanical behavior and corrosion resistance in biological surroundings [1,2]. However, there are still challenging problems to solve, such as bone resorption of tissues adjacent to the implant, related to the phenomenon of stress-shielding [3], as well as implant loosening caused by poor osseointegration and/or bacteria proliferation. In this context, the use of β-titanium alloys [4,5,6,7] and porous titanium implants [8,9,10] have been widely recognized as valid approaches for eliminating the effect of stress-shielding on titanium implants. The latter also allows proper vascularization through the interconnected pores, for desirable bone in-growth [11]. Furthermore, good implant osseointegration implies adequate adhesion, proliferation, and differentiation of bone tissue cells have been achieved on its surface [12,13].

Several methods were proposed to improve the osseointegration of prosthetics, mainly based on surface, chemical, or physical modifications. On the one hand, chemical techniques alter the composition of implant surfaces by coating, impregnation, immersion, or deposition of bioactive glasses, ceramics, polymers, or peptides [1,14,15,16]. On the other hand, among the techniques to modify the texture and roughness of the implant surface, it is worth highlighting sand- and grit-blasting, acid-etching, ultraviolet treatment, electrochemical anodizing, spark anodizing, direct irradiation synthesis methods, and laser surface modification [17,18,19,20,21,22,23,24]. The main goal of physical modifications of the surface of Ti and Ti-alloy implants is the creation of micro- and nano-structures to stimulate osseointegration [25] by increasing the porosity for cell adhesion and proliferation or adapting roughness to better wettability, protein adsorption, and bactericidal response. Furthermore, the high roughness in terms of patterned surfaces is also suitable for preventing bacterial colonization [26,27,28].

Recently, laser surface modifications were exponentially employed due to their superior advantages over other physical techniques such as more accurate control of specific topological designed features on the surface, high efficiency, and low material consumption [29]. In particular, femtosecond laser (FSL) ablation of Ti surfaces has been widely investigated in the past two decades as this technique allows for high precision and control of desired patterns on the surfaces, as well as it being low cost and having a reliable process [24,30]. A wide variety of micro- and nano-structures [29,31] can be designed on Ti surfaces depending on the FSL beam parameters [31,32,33,34,35]. Different authors, [36,37,38], have validated the improvement of biocompatibility of Ti and Ti-alloy surfaces using the FSL technique, generating bioinspired micro- and nano-features such as laser-induced periodic surface structure (LIPSS), ripples, columns, pits, or spikes among others [39,40]. In particular, Liang et al., [41] demonstrated better osseointegration and cell proliferation of pure Ti implants using FSL ablation combined with Ca/P deposition. The formation of a micro-pattern on the Ti surface helped in accelerating the cellular integration compared with those of pure Ti and sand-blasted Ti implants. Furthermore, Wang et al., [42] have demonstrated the capabilities of micro-grooved Ti6Al4V implants by FSL and chemical assembly of graphite (G) and graphene oxide (GO), which could improve cell adhesion, proliferation, and osteogenic differentiation and also induce surface wettability and bone-like apatite formation.

However, an appropriate roughness of metallic implant surfaces could allow for the control of wettability [43] and therefore enhance the hydrophilicity or hydrophobicity [44,45] of these surfaces. This aspect is very interesting to prevent bacterial adhesion and biofilm formation [9]. In this regard, Cunha et al. [46] tested the adhesion and biofilm formation of *Staphylococcus aureus* on surfaces of FSL-patterned titanium alloys compared to polished ones. The nano topography size of single features and the distance between them induce a significantly reduced contact area interface between the individual bacterium and the metal to make bacteria agglomeration difficult for ulterior biofilm formation.

Few works have reported on the study of surface modification of porous titanium implants. In particular, the authors of this work have performed preliminary studies in which hierarchical micro- and nano-structures such as micro-holes, micro-columns, and laser-induced periodic surface structure (LIPSS) could be created on porous titanium surfaces. Despite these promising results, understanding the phenomena that occur on femtosecond laser-modified surfaces remains a challenge [47]. Therefore, this current work studies how the macro-porosity (percentage and range size) of titanium substrates influences the final surface roughness generated by laser irradiation, as well as its relationship with the tribo-mechanical behavior, wettability of the surface, and the in vitro cellular response.

## 2. Materials and Methods

### 2.1. Substrates Preparation

**Figure 1:**
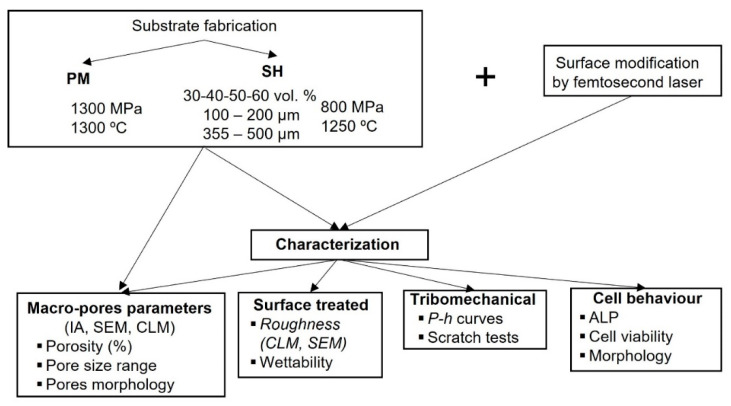
Scheme of the procedure followed by sample fabrication, modification, and characterization. Abbreviations: PM (Pulvimetallurgy), SH (Space Holder), IA (Image Analysis), SEM (Scanning Electron Microscopy), CLM (Confocal Laser Microscopy), ALP (Alkaline Phosphatase) and *P-h* (micro-indentation curve Power or Force applied vs. penetration deep).

Figure 1 shows a diagram of the procedure followed by substrate fabrication, superficial modification, as well as tribo-mechanical and in vitro cell characterization. Commercially pure Ti (C.p. Ti), (SEJONG Materials Co. Ltd. Seoul, Korea) with a chemical composition according to the standard [48] and a particle size distribution d_(50)_ = 23.3 µm and d_(90)_ = 48.8 µm [49] was employed to fabricate the fully-dense discs as reference samples, as well as porous substrates, using powder metallurgy technology (PM). Fully-dense samples were fabricated by pressing at 1300 MPa and then sintering at 1300 °C. Porous samples were manufactured using the space holder technique (SH) with ammonium bicarbonate (NH_4_HCO_3_) (Cymit Quimica S.L., Barcelona, Spain) as spacer particles in different volume percentages (30, 40, 50, and 60 vol.%) and range sizes (100–200 and 355–500 µm). C.p. Ti was mixed with the corresponding amount of spacer particles (percentage and range size), pressed at 800 MPa, and then sintered at 1250 °C for 2 h in a high vacuum atmosphere (~10^−5^ mbar). The spacer particles were removed before sintering in two stages (60 °C and 110 °C) for 12 h each. Before laser modification, the surface of the discs was carefully ground and polished to preserve the porosity fraction, size, and morphology of the pores.

Next, both types of titanium substrates were subjected to surface modification using a femtosecond Yb-doped fiber laser (Spirit 1040–4, Spectra-Physics, Santa Clara, CA, USA). The laser system generates 396 fs pulses with a maximum pulse energy of Ep = 49.7 µJ, at a repetition rate of f = 100 kHz. A computer-controlled galvanometric scanning system was used to direct the laser beam across the target surface. A flat field lens kept the laser focused on the surface to a spot with radius w0 = 12 µm. The irradiation was carried out along parallel lines at a constant speed of v = 960 mm/s in the scan direction and the laser paths were laterally overlapped with an overlap of s = 50% until the entire surface of the workpiece was processed. The surface was irradiated 20 times after this procedure, resulting in 100 accumulated laser pulses per spot and fluency of F = 21.98 J/cm^2^. Experiments were performed in air using an Ar jet to reduce surface oxidation These parameters were selected after performing preliminary tests with the goal of obtaining a hierarchical surface structure consisting of both laser-induced micro-structures and laser-induced periodic surface structures (LIPSS) at the nano-metric level.

The modification of the surface due to the femtosecond laser treatment was evaluated by scanning electron microscopy (SEM), (FEI TENEO, Eindhoven, The Netherlands) and confocal laser microscopy (CLM), (Sensofat S Nexox; Barcelona, Spain). CLM allowed the acquisition of two-dimensional (2D) and three-dimensional (3D) images and parameters related to roughness such as the arithmetical mean deviation (*S_a_*) and the root-mean-square height (*S_q_*). Furthermore, the percentage of total porosity (*P_T_*) and the equivalent diameter of the pores (*D_eq_*) of the surface (before and after femtosecond treatment) were evaluated, using SEM images and Image-Pro Plus 6.2 software (Rockville, MD, USA).

Wettability was evaluated by static contact angle (CA) measurements obtained with an OCA 20 (Data Physics Instruments GmbH, Filderstadt, Germany) goniometer set up by depositing macroscopic droplets on the surface of the samples according to Young’s method. Measurements were based on a minimum of 3 data points per sample, taking the average as the CA value. Totals of 2 µL bidistilled water (pH 7) and 5 µL bovine serum albumin (Merck Life Science S.L.U., Madrid, Spain) droplets were used.

### 2.2. Tribo-Mechanical Characterization of Modified Substrates

First, macro-mechanical behavior of the porous c.p. Ti substrates (yield strength, *σ_y_*, and dynamic Young’s modulus, *E_d_*) were estimated from porosity data, using equations already reported in the literature [50]. For these equations, Young’s modulus for bulk c.p. Ti grade IV was ~ 110 GPa [51] and the yield strength of the bulk c.p. Ti grade IV was ~650 GPa [52]. The micro-mechanical characterization and scratch-resistance of the surface of modified substrates were evaluated using instrumented micro-indentation (*P–h* curves) and scratch tests, respectively. Static loading–unloading tests were performed on a MICROTEST machine (Microtest Company, Madrid, Spain). A preload of 0.05 N was used to ensure contact between the Vickers indenter and the surface, 0.9 N being the maximum load, which was applied with a rate of 0.5 N/min and a dwell time of 40 s. The micro-hardness and Young’s modulus were calculated from these data applying Oliver and Pharr method [53]. Additionally, scratch resistance was measured using the same commercial MICROTEST device with a constant applied load of 3 N at a rate of 0.5 mm/min for a scar of 3 mm, using a Rockwell diamond tip of 200 µm of diameter. Variation width was recorded with applied load of the in situ penetration depth and permanent plastic deformation depth. In addition, elastic recovery of the material and the damage inherent to the imposed tribo-mechanical stresses were also evaluated by SEM and CLM.

### 2.3. Cellular Behavior of Modified Surfaces by Femtosecond Laser Texture

Finally, cell behavior was evaluated in terms of growth, proliferation, and morphology using different techniques.

### 2.4. In Vitro Cell Culture Techniques

MC3T3-E1, a murine pre-osteoblast cell line (CRL-2593 from the American Type Culture Collection (ATCC), Manassas, VA, USA), was used to analyze the possible influence of surface modified with FSL on bone cells. All c.p. Ti substrates (fully-dense and porous with different percentages of porosity and pores range size) were tested for cell metabolism and viability during the cell adhesion, proliferation, and differentiation process.

### 2.5. Cell Culture

Routine cell line passaging was performed in 100 mm plates with Minimum Essential Medium (αMEM), containing 10% fetal bovine serum (FBS) plus antibiotics (100 U/mL penicillin and 100 mg/mL streptomycin sulfate) (Invitrogen, Carlsbad, CA, USA). The discs were autoclaved at 121 °C for 30 min and then placed on a 24-well plate. Osteoblast cells were seeded at a cellular density of 35,000 cells/cm^2^. Plates were kept at 37 °C and 5% CO_2_ atmosphere. Fully dense c.p. Ti discs were used as a reference.

At 48 h of osteoblast culture, they were induced to undergo differentiation using osteogenic induction medium consisting of α-MEM medium, 10% Fetal Calf Serum (FCS), 10 mM ascorbic acid (Merck, Darmstadt, Germany), and 50 µg/mL of β-glycerophosphate (StemCell Technologies, Vancouver, BC, Canada). The medium was replaced every 2 days. The in vitro cell experiments were carried out at 21 days.

### 2.6. Cell Viability and Proliferation Assay

Cell proliferation and viability tests were evaluated using AlamarBlue^®^ reagent (Invitrogen, Carlsbad, CA, USA). According to the manufacturer’s protocol. Subsequently, the absorbance at 570 nm (oxidized) and 600 nm (reduced) (TECAN, Infinity 200 Pro, Männedorf, Switzerland) was recorded.

### 2.7. Cell Differentiation by Alkaline Phosphatase (ALP) Evaluation

MC3T3 differentiation levels were evaluated by alkaline phosphatase (ALP) activity, using the Alkaline Phosphatase Assay Kit (Colorimetric) (Abcam, Cambridge, UK). The assay was performed in triplicate according to the manufacturer’s protocol. The absorbance at 405 nm of 4-nitrophenol was measured in a 96-well microplate reader. Data were expressed as µmol/min/mL of *p*-Nitrophenyl Phosphate (*p*NPP).

### 2.8. Cell Morphology

Scanning electron microscopy images acquired with a Zeiss EVO LS 15 scanning electron microscope (SEM), (Zeiss, Oberkochen, Germany) with an acceleration voltage of 10 kV were used to evaluate cell behavior at 21 days. The samples were fixed in 10% formalin, followed by a dehydration step with ethanolic solutions, and then coated with gold-coating using a sputter coater (Pelco 91000, Ted Pella, Redding, CA, USA) before the SEM study.

### 2.9. Statistical Analysis

All experiments were carried out in triplicate to ensure reproducibility. The results were expressed in terms of mean and standard deviation to perform a two-way ANOVA followed by Tukey’s post-test using SPSS v.22.0 for Windows (IBM Corp., Armonk, NY, USA). The significance level was considered at *p*-values of *p* ˂ 0.05 (*) and *p* ˂ 0.01 (**).

## 3. Results and Discussion

**Figure 2:**
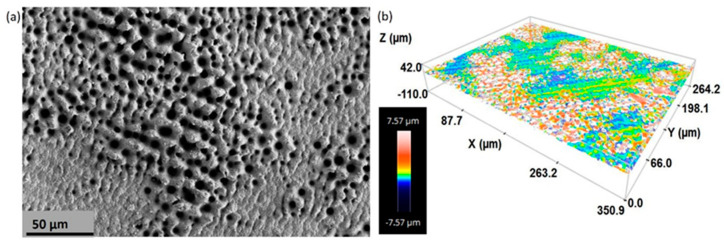
(**a**) SEM (Scanning Electron Microscopy) and (**b**) 3D-CLM (Confocal Laser Microscopy) images of the fully-dense c.p. Ti substrates after femtosecond laser treatment.

**Figure 3:**
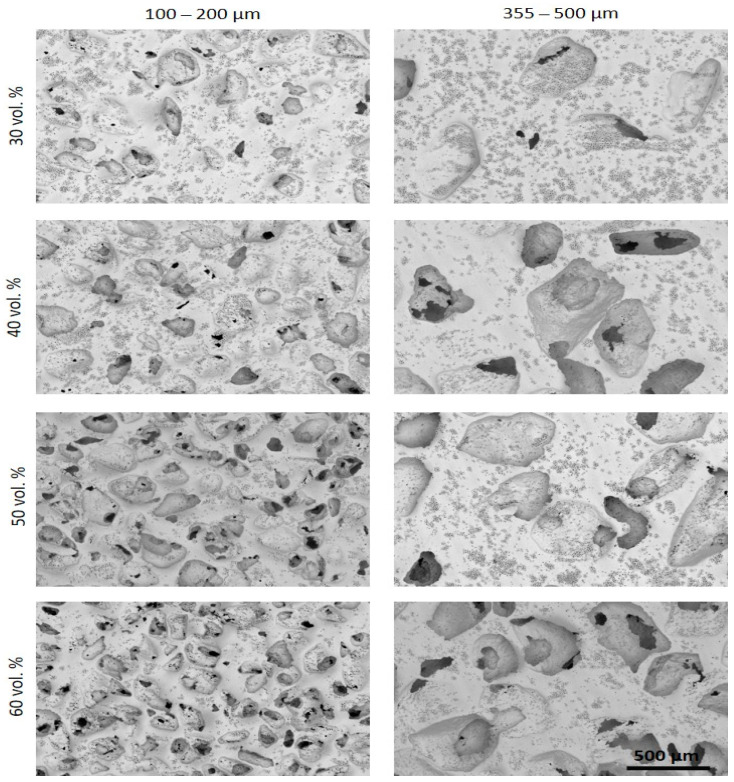
SEM images after the femtosecond laser treatment of the different porous c.p. Ti substrates. Images acquired using the topography view configuration gather both material and topographic contrast with the unique segmented in-lens backscattered electron detector (BSE). Common scale bar for all subfigures.

**Figure 4:**
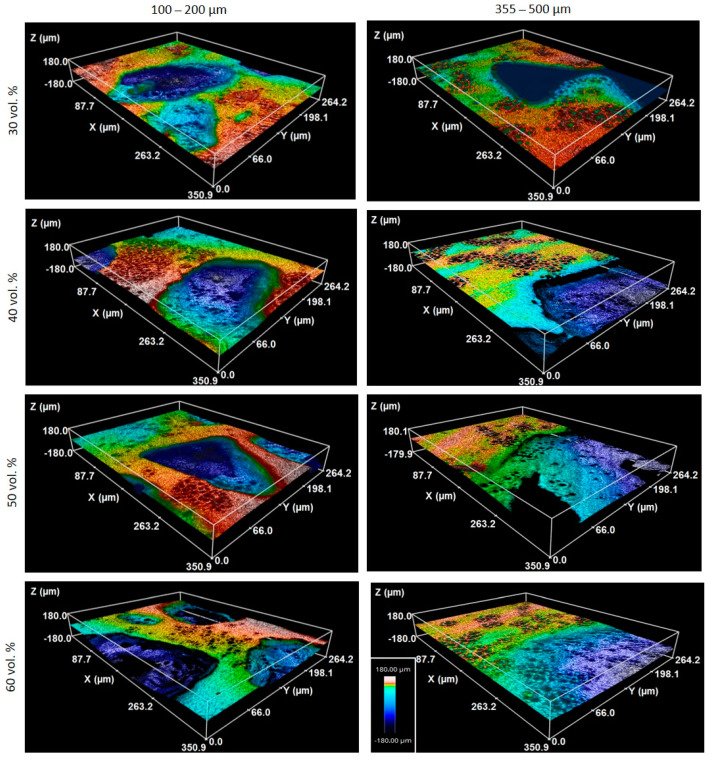
CLM images after the femtosecond laser treatment of the different porous c.p. Ti substrates. Common scale bar for all subfigures.

SEM (Figure 2a and Figure 3) and CLM (Figure 2b and Figure 4) images acquired after femtosecond laser treatment displayed the formation of a rough surface with micro-columns and micro-holes for all the studied substrates. The surface was covered with a pattern formed by nano-metric ripples over the micro-metric structures. These laser-induced ripples were periodic surface structures that appeared when the surface was subjected to ultra-short laser pulses. Moreover, the periodic ripples were aligned perpendicularly to the polarization of the laser beam. In the case of porous substrates, the pores resulting from the spacer particles were also observed. In the case of the fully-dense samples, the generation of a roughness pattern on the surface of the substrates was clearly observed (Figure 2a) with the formation of micro-holes and micro-columns.

In the case of the porous c.p. Ti substrates (Figure 3 and Figure 4) independently of the pore size and percentage of porosity, the results were also evident in terms of the formation of these micro-structures, micro-columns, micro-holes, and ripples, among the pores generated by the spacer particles. The structures generated by the laser appeared both in the flat area and inside the macro-pores. In fact, there was no appreciable difference between the textures in the flat areas and the inner surface of the macro-pore walls. From the SEM micrographs (Figure 3), macro- and micro-porosity measurements were performed and parameters relating to the macro-mechanical behavior were estimated based on the porosity data (Table 1). The analyses of these results are shown in Table 1 and allowed us to indicate that: (1) three types of pore populations were identified: micro-pores inherent to the sintering of the substrates, micro-columns resulting from the femtosecond treatment, and macro-pores associated with the spacer particles; (2) an inverse relationship between porosity (percentage, size, and degree of interconnectivity) and the macro-mechanical behavior (Young’s modulus and yield strength) of the studied materials was corroborated; and (3) it was also observed that the number and diameter of the micro-columns depended on the size of the titanium matrix that was modified by femtosecond laser radiation. This tendency of the micro-columns might be related to the heat evacuation phenomena during the interaction between the laser radiation and the titanium matrix.

**Figure 5:**
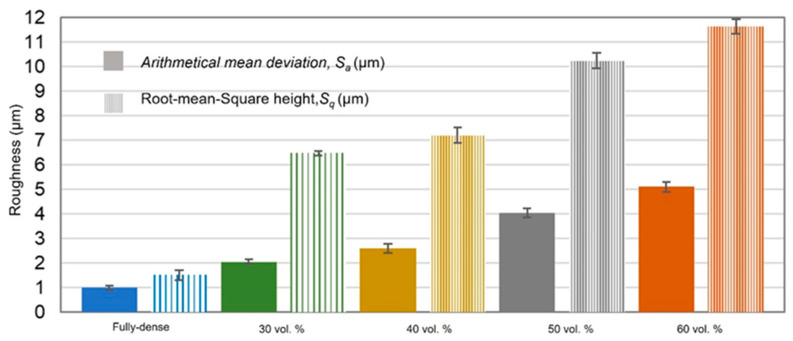
*Sa* and *S_q_* values measured from the CLM images for all the studied c.p. Ti substrates (fully-dense and 100–200 µm for porous substrates).

Furthermore, analysis of CLM images allowed us to measure the roughness parameters due to superficial modification of the substrates (Figure 5). For porous substrates, the roughness of the flat area among the pores was also measured. Before laser treatment, the fully-dense sample presented very low roughness, while the porous sample had slightly higher roughness due to the existence of pores (all substrates were previously mirror-polished). After laser irradiation, the roughness of all the samples increased. The changes in topography can be clearly seen in Figure 2 and Figure 4. Laser radiation produced a surface with heterogeneous heights and a grainy texture. A relationship was also found between the porosity of the substrates and the final roughness, a higher increase in the surface roughness after laser irradiation was observed for highly porous substrates, as shown in Figure 3, Figure 4 and Figure 5. It is evident that the Sa increases as the number of pores per unit area increases.

**Figure 6:**
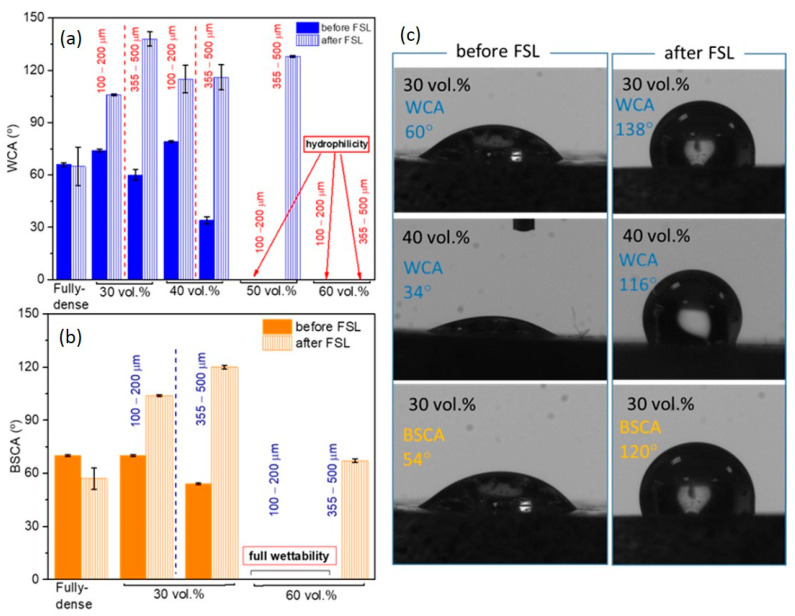
Wetting analysis of the porous c.p. Ti substrates: (**a**) Water Contact Angle (WCA) values of surfaces with different porosity percentages and pores range size before and after the FSL (Femto Second Laser) treatment; (**b**) bovine serum contact angle (BSCA) values of porous surfaces before and after the FSL treatment; (**c**) images representing the wetting of water and bovine serum droplets deposited on c.p. Ti porous surfaces (355–500 µm pores range size) before and after FSL treatment.

The fully-dense surface showed a partial hydrophilic behaviour with water contact angles around 65° (Figure 6). The substrates prepared with smaller spacer particles tended to have a hydrophobic character, while larger pores induced a notable decrease in the water contact angle that agreed with the Wenzel model [54]. Thus, as the surface roughness and pore size increased, the surfaces with the highest porosity reached a completely hydrophilic character.

The first interaction of these porous c.p. Ti surfaces with a simulated physiological fluid was tested through the study of wettability with bovine serum albumin. In that case, larger pores were demonstrated to improve the wettability of the surfaces when compared to a fully-dense surface and even to those with smaller pore sizes. In general, after FSL treatment, the comparison of the water contact angle and the values of *S_a_*, previously described in Figure 5, showed that for a roughness acquired with 30 vol.% and larger pores samples, hydrophobicity was promoted. This repulsive behaviour was also manifested when bovine serum droplets were deposited on the porous surfaces, reaching contact angle values greater than 100°. Special mention was deserved for the substrates with 60 vol.%, which went from being completely wet to maintaining a contact angle of over 70°. These results pointed to a remarkably stable and protective response when exposed to the action of the biological environment, as well as the expected antibacterial behavior of the surface, which prevented the generation of spores and bacterial contamination on the surface of implants [55,56]. Cell adhesion was not only affected by wettability but roughness and surface chemistry also play competitive roles, plus differences in the sizes and elastic responses of bacterial cells compared to bone tissue cells were added factors [57] that would allow the hydrophobic porous c.p. Ti surfaces to selectively induce a reduction in the bacteria attachment in favor of a greater living cell proliferation [58,59].

**Figure 7:**
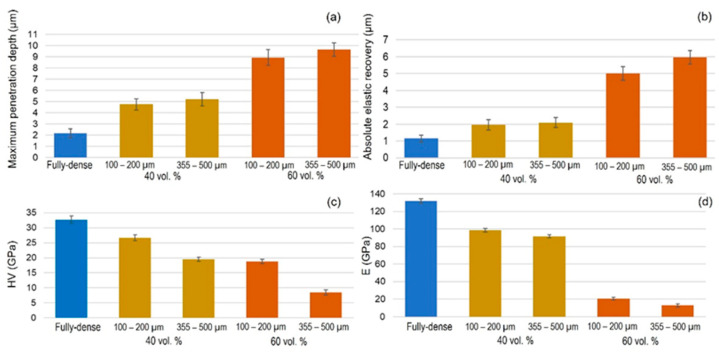
Static loading–unloading test (*P–h* curves) parameters of the different FSL modified substrates studied: (**a**) maximum penetration depth; (**b**) absolute elastic recovery; (**c**) micro-hardness (*HV*); and (**d**) Young’s modulus (*E*). Note: c and d are calculated following the Oliver and Pharr method.

**Figure 8:**
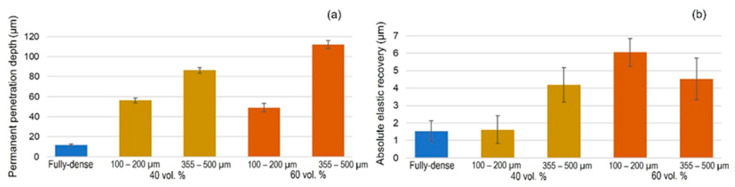
Scratch tests parameters on FSL-modified titanium substrates: (**a**) permanent penetration depth; and (**b**) absolute elastic recovery (difference between scratch in situ tests and permanent penetration depth).

**Figure 9:**
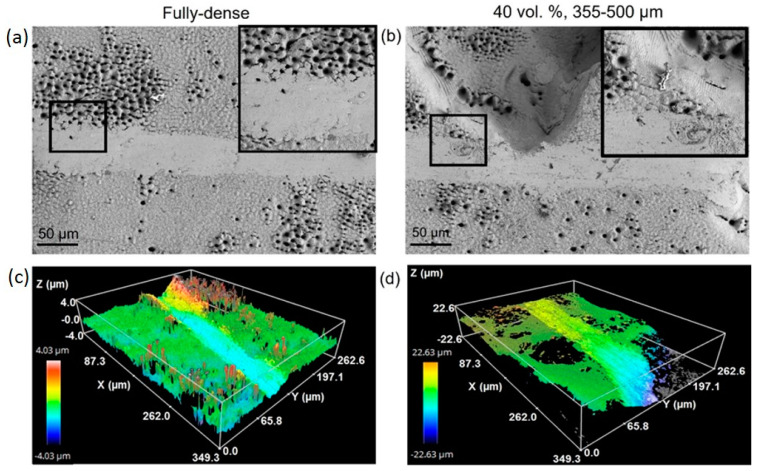
(**a**,**b**) SEM and (**c**,**d**) CLM images of scar (due to the scratch test) on fully-dense and porous c.p. Ti substrates. Inset SEM: zoomed details of area marked in subfigures.

Figure 7 and Figure S1 display the most relevant results inherent to the instrumented micro-indentation tests (*P–h* curves, static behavior). On the other hand, in Figure 8 and Figure S2 the results of the scratch tests (dynamic characterization) are showne. Both figures compare the fully-dense substrate to the 40 vol.% and 60 vol.% porous substrates for both ranges of analyzed pore sizes. A higher penetration depth was observed for higher porosity (60 vol.%), almost independently of the pore size. Furthermore, mechanical properties such as the micro-hardness and Young’s modulus were estimated from the resulting *P–h* loading and unload curves using the Oliver and Pharr method described above. In general, the micro-hardness, Young´s modulus, and scratch resistance decreased as the pore size and porosity increased. Regardless of the type of test, static (*P–h*) and dynamic (scratch test), the elastic recovery is proportional to the porosity of the titanium substrate. Figure 9 shows SEM and CLM images (2D and 3D) of the grooves generated by the scratch tests. As expected, the width and depth of the scar were inversely proportional to the resistance to scratching of the surfaces. An additional widening inherent to the presence of the macro-pores was observed, as well as a collapse (plastic deformation) of the micro-columns.

### 3.1. Cell Viability and Proliferation Study

**Figure 10:**
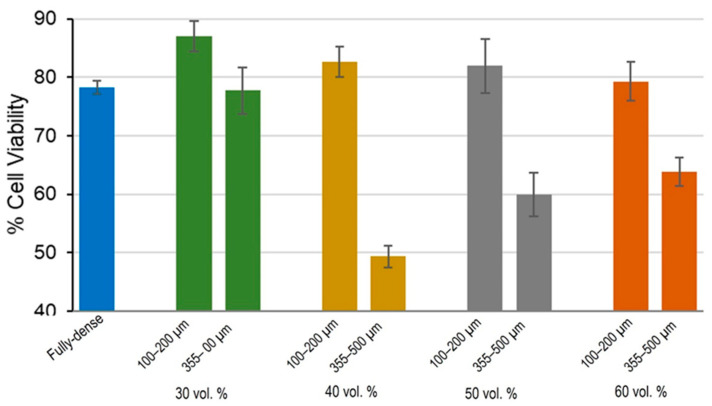
Effects of the percentage of porosity and pores range size on cell viability in MC3T3–E1 cells. Results are represented as % cell viability.

The study of the degree of proliferation and viability on modified laser surfaces reached at 21 days revealed the best results for 30 vol.% substrates, where porosity increased and cell proliferation decreased slightly (see results in Figure 10). When comparing cell growth according to pore size, it was found that the smallest pore size range was the most favorable condition for osteoblast cell growth, while growth over substrates with larger pores showed worse proliferation. That is, on porous substrates with pores of 100–200 µm, cells proliferated better than in the case of larger pore sizes. Only in discs with 30 vol.% porosity and 100–200 µm pore size, cell growth was greater than 80%. The other porous substrates presented values similar to those of the fully-dense c.p. Ti substrates. Regarding the degree of porosity, the 30 and 40 vol.% samples showed better cell growth, although the differences were not significant (Figure 10). These results confirmed the affinity of osteoblast cells for growth on a surface modified by FSL [45,47,60] and, as porosity increased, the contact surface and growth also increased [61]. In addition, as already reported in the literature [62], in this work it was also observed that 100–200 µm pore sizes that are more similar to cell area size were more favorable for monolayer growth of osteoblasts.

### 3.2. Cell Functional Activity

**Figure 11:**
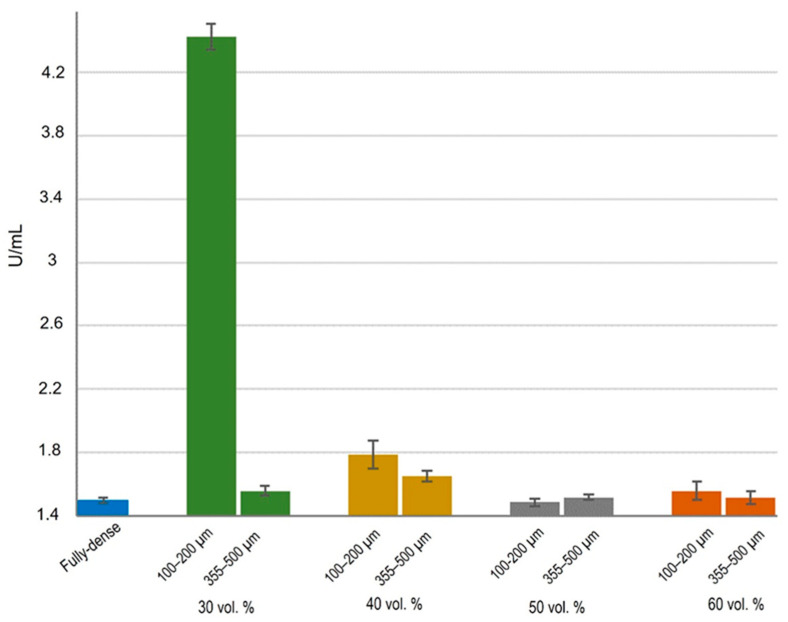
Cell differentiation of osteoblasts, in vitro evaluation of alkaline phosphatase enzyme (ALP) activity measured as U/mL. Statistical differences are indicated at * *p* < 0.05.

ALP activity was used to assess improved osseointegration capacity as markers of early differentiation of osteoblast-like cells [63]. The activity of ALP was observed to be similar in all cases (Figure 11), except for samples with 30 vol.% porosity and a smaller pore range size. Under this condition, osteoblasts showed activity more than double that of the other substrates. At the metabolic level, osteoblasts grown in 30 vol.% and 100–200 µm samples showed greater cellular functionality compared to other conditions, therefore there were more differentiated cells. No differences in the rest of the conditions were found, either with porosity percentage and/or with pore size range. Other authors also showed higher ALP values in MC3T3 cell cultures on FSL-treated surfaces [64,65]. In view of these results, it could be suggested that the better proliferation was due to the size of the osteoblast cells, which allowed them to create better cell–cell interactions on this type of surface and consequently, greater stimulation in cell growth and differentiation.

### 3.3. Morphological Study by SEM

**Figure 12:**
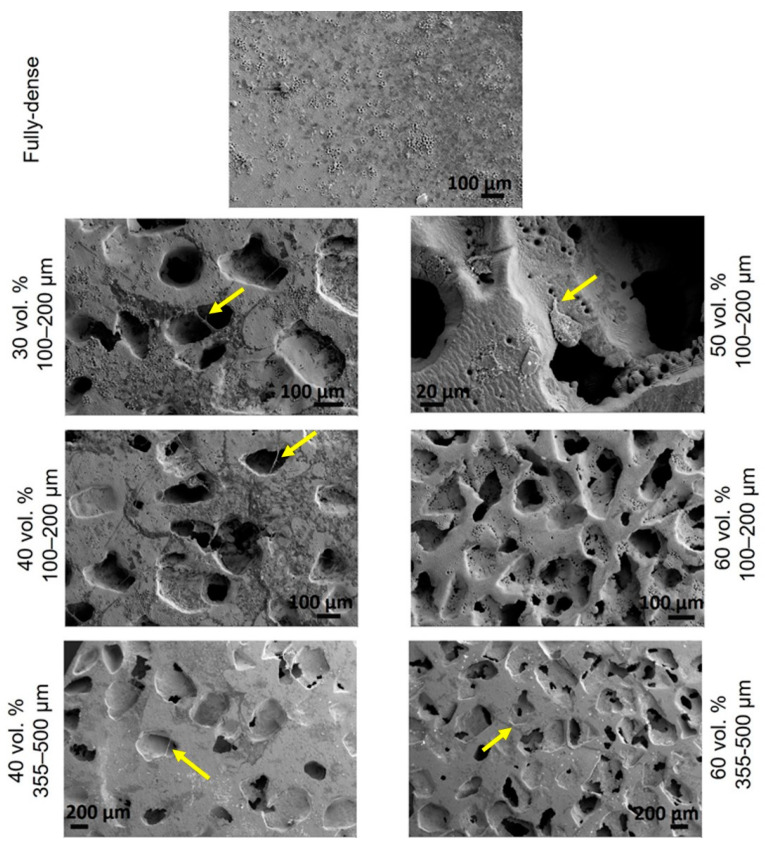
SEM images of the cell adherence of the fully-dense and porous substrates after femtosecond laser treatment. Cell–cell interactions and filopodium (yellow arrow) are indicated in the images.

The spread of MC3T3-E1 cells was observed in each studied condition by SEM images. As shown in Figure 12, osteoblasts showed a characteristic growth direction, although growth was lower for substrates with the largest pores, in which it was observed that cells could not grow in a monolayer throughout the surface. In samples with smaller pores, cell growth was observed both outside and inside the pores, there were even filopodium-like union structures among cells located on the periphery of the pores [60,66]. In general, the cells presented an elongated shape with slightly pronounced outstretched filopodium. Specifically, for samples with 30 and 40 vol.% porosity and pore sizes in the range of 100–200 µm, a more fibroblastic cell shape was observed, indicating a higher degree of differentiation, which was consistent with the measured ALP values.

## 4. Conclusions

In summary, in this work, micro-structural, tribo-mechanical characterization, and in vitro cellular behavior evaluations were performed on fully-dense and porous c.p. Ti samples that were superficially FSL-modified. The use of femtosecond laser radiation increased the surface roughness for all studied samples, generating characteristic textures and micro-columns. Treatment with FSL generated notably hydrophobic porous surfaces, with an increase in the water and bovine serum contact angle values as the pore size increased, as shown in the cases of 30, 40, and 50 vol.%, indicating a potential protective and antiseptic behavior on the surface of the implants working in a biological environment, preventing bacterial proliferation and dissemination without any detrimental effect on biocompatibility. In tribo-mechanical terms, Young´s modulus and scratch-resistance were found to decrease as the pore range size and the percentage of porosity increased, while the elastic recovery was directly proportional to the porosity of the titanium substrate. Surface modification with femtosecond laser treatment improved cell viability by 14%, mainly in the case in which osteoblasts were grown with 30 vol.% and a 100–200 µm pore size substrate, which showed better cell differentiation potential, proliferation, and viability performance.

## Figures and Tables

**Table 1:** Experimental porosity parameters and estimated macro-mechanical behavior of porous implants before and after FSL. Note: Flat surface (between macro-pores).

Discs Tested			Before FSL					After FSL		
			*P_T_* (%)	*D_eq_* (μm)	*E_d_* (GPa)	*σ_y_* (MPa) *		*P_T_* (%)	*D_eq_* (μm)	*E_d_* (GPa) **
Conventional PM			1.6 ± 0.3	3.9 ± 0.2	103.2 ± 1.3	739 ± 6		25.6 ± 0.9	6 ± 1.2	62.6 ± 1.2
Space-holder technique	30 vol.%	100–200 μm	30.2 ± 0.2	192 ± 117	56.8 ± 0.7	354 ± 26	P_F_ ***	9.8 ± 0.7	10 ± 3	50.2 ± 1.5
							P_SH_	27.2 ± 0.9	111 ± 2	
		355–500 μm	30.1 ± 0.1	335 ± 40	57.0 ± 1.4	341 ± 37	P_F_ ***	9.4 ± 0.8	11 ± 3	44.6 ± 1.1
							P_SH_	32.2 ± 0.9	250 ± 5	
	40 vol.%	100–200 μm	40.2 ± 1.1	226 ± 178	45.9 ± 1.2	234 ± 28	P_F_ ***	6.7 ± 1.1	11 ± 3	35.7 ± 1.0
							P_SH_	45.4 ± 0.6	138 ± 3	
		355–500 μm	40.8 ± 1.3	359 ± 123	45.3 ± 1.1	206 ± 26	P_F_ ***	6.4 ± 0.9	12 ± 3	39.7 ± 1.2
							P_SH_	40.3 ± 1.2	322 ± 4	
	50 vol.%	100–200 μm	52.3 ± 1.2	164 ± 28	35.4 ± 1.9	95 ± 30	P_F_ ***	5.8 ± 1.5	12 ± 4	30.0 ± 1.0
							P_SH_	56.3 ± 1.1	197 ± 3	
		355–500 μm	50.1 ± 1.0	365 ± 34	37.1 ± 1.6	118 ± 22	P_F_ ***	3.8 ± 1.3	11 ± 4	35.4 ± 1.4
							P_SH_	46.5 ± 1.4	340 ± 5	
	60 vol.%	100–200 μm	56.4 ± 0.5	189 ± 105	32.3 ± 1.5	91 ± 27	P_F_ ***	2.7 ± 1.0	9 ± 2	26.5 ± 1.4
							P_SH_	63.6 ± 0.9	205 ± 4	
		355–500 μm	57.8 ± 0.6	395 ± 131	31.3 ± 1.6	84 ± 31	P_F_ ***	2.5 ± 1.3	8 ± 2	25.4 ± 1.6
							P_SH_	64.5 ± 1.5	325 ± 2	

* The values of the yield stress estimated before and after femtosecond laser treatment were similar because the static mechanical behavior does not depend on the additional micro-porosity generated on the surface of the samples by the laser. ** The estimated *E_d_* values after FSL corresponded to the surface of the samples (influences the additional porosity due to surface treatment). *** Micro-porosity generated with the femtosecond laser relative to the effective area of the titanium matrix between the pores.

## Data Availability

All the data is available within the manuscript.

## Supplementary Materials

The following supporting information can be downloaded at: https://www.mdpi.com/article/10.3390/ma15092969/s1, Figure S1: *P*–*h* curves of the study of substrates modified by femtosecond and calculated mechanical properties based on Oliver and Pharr method; Figure S2: Resistance to penetration evaluated by scratch tests, for the pore range size 100–200 µm and two porosities; Figure S3: Scanning electron microscopy image of the scar on a substrate of 60% vol% and pore range size 355–500 µm.
