# Supplementary material for: Influence of Femtosecond Laser Modification on Biomechanical and Biofunctional Behavior of Porous Titanium Substrates
Source: Materials (Basel). 2022 Apr 19;15(9):2969. doi: 10.3390/ma15092969 (PMC9099494; doi:10.3390/ma15092969)
Supplement: Supplementary file 1 [file materials-15-02969-s001.zip › materials-1665489-supplementary.pdf]

Influence of Femtosecond Laser Modification on Biomechanical and Biofunctional Behavior of Porous Titanium Substrates

Ana M. Beltrán [1], Mercè Giner [2], Ángel Rodríguez [3,*], Paloma Trueba [1], Luisa M. Rodríguez-Albelo [1,*], M. Angeles Vázquez-Gámez [4], Vanda Godinho [1], Ana Alcudia [5], José M. Amado [3], Carmen López-Santos [6,7] and Yadir Torres [1]

[1] Departamento de Ingeniería y Ciencia de los Materiales y del Transporte, Escuela Politécnica Superior, 41011, Universidad de Sevilla, Seville, Spain; abeltran3@us.es (A.M.B.); ptrueba@us.es (P.T.); vfortio@us.es (V.G.); ytorres@us.es (Y.T.)
[2] Departamento de Citología e Histología Normal y Patológica, e, Universidad de Sevilla, Seville, Spain;
mginer@us.es
[3] Departamento Ingeniería Naval e Industrial, Escuela Politécnica Superior, Campus Industrial, Universidade da Coruña, 15403, Ferrol, Spain; jose.amado.paz@udc.es
[4] Departamento de Medicina, Universidad de Sevilla, Seville, Spain; mavazquez@us.es
[5] Departamento de Química Orgánica y Farmacéutica, Facultad de Farmacia, 41005, Universidad de Sevilla, Seville, Spain; aalcudia@us.es
[6] Departamento de Física Aplicada I, Escuela Politécnica Superior, 41011, Universidad de Sevilla, Seville, Spain. mlopez13@us.es
[7] Nanotecnología en Superficies y Plasma, Instituto de Ciencia de Materiales de Sevilla,41092, Seville, Spain
* Correspondence: angel.rcarballo@udc.es (Á.R.); lralbelo@us.es (L.M.R.-A.)

Supplementary information

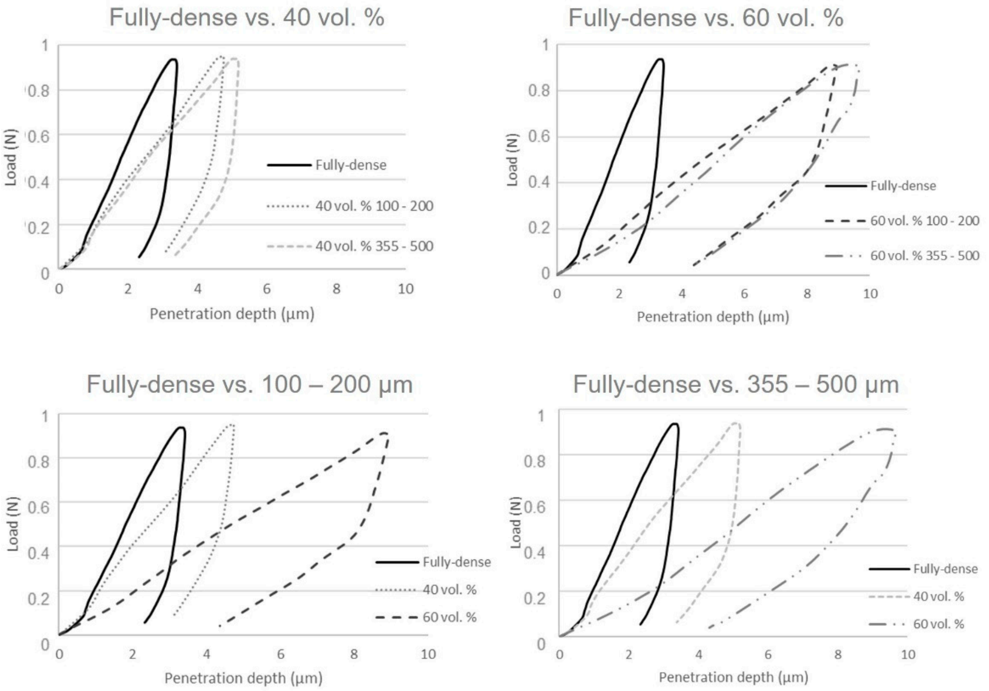

		HV (GPa)	E(GPa)
Fully-dense		32.7 ± 1.2	131.9 ± 2.7
40 vol. %	100 – 200 µm	26.6 ± 1.0	98.5 ± 2.0
	355 – 500 µm	19.5 ± 0.7	91.5 ± 1.8
60 vol. %	100 – 200 µm	18.8 ± 0.7	20.5 ± 1.5
	355 – 500 µm	8.5 ± 0.8	12.8 ± 1.8

Figure S1. *P-h* curves of the study substrates modified by femtosecond and calculated mechanical properties based on Oliver and Pharr method.

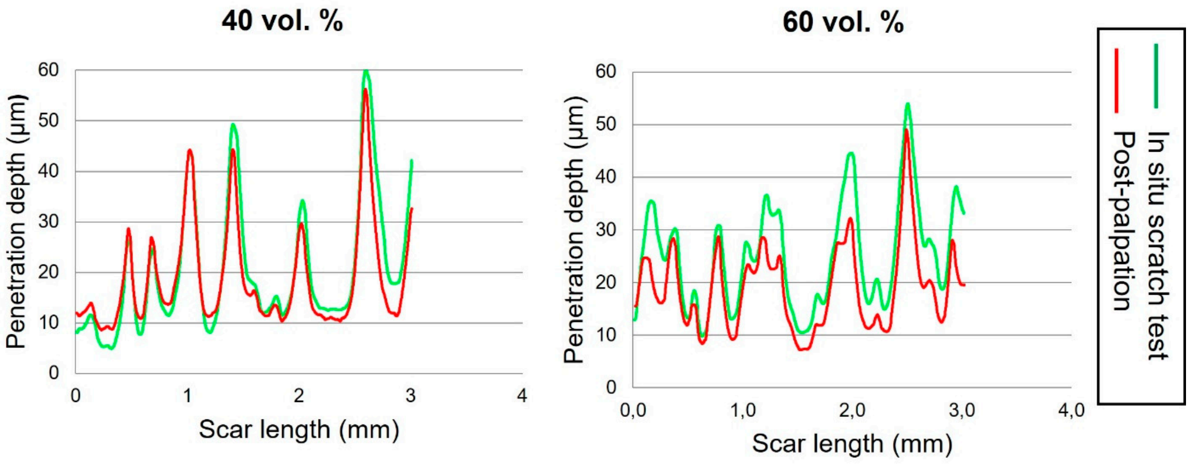

Figure S2. Resistance to penetration evaluated by scratch tests, for the pore range size 100 – 200 µm and two porosities.

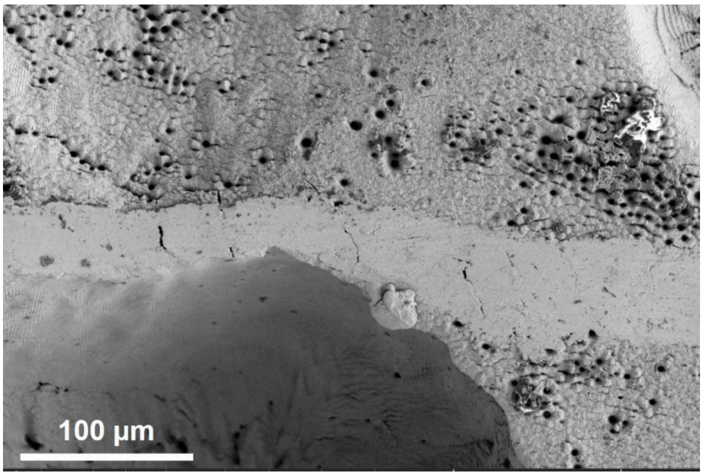

Figure S3. Scanning electron microscopy image of the scar on a substrate of 60 vol. % and pore range size 355 – 500 µm.
